# Delayed PCI is not beneficial for STEMI patients with impaired renal function: a retrospective cohort study

**DOI:** 10.1186/s12872-023-03271-2

**Published:** 2023-05-19

**Authors:** Yi Lao, Kaitong Chen, Li Feng, Yong Yuan, Jin Zhang, Liting Zhang, Xuansheng Huang, Mingxing Li, Zidi Wu, Jianping Bin, Yulin Liao

**Affiliations:** 1 Department of Cardiology, State Key Laboratory of Organ Failure Research, Guangdong Provincial Key Laboratory of Heart Function and Microcirculation, Nanfang Hospital, Southern Medical University, Guangzhou, Guangdong province China; 2 Cardiovascular Department, The First Affiliated Hospital of Guangdong Pharmaceutical University, Guangzhou, Guangdong Province China; 3 Department of Cardiology, Zhongshan Hospital, Sun Yat-sen University, Zhongshan City, Guangdong province China

**Keywords:** Acute myocardial infarction, Percutaneous coronary intervention, Late reperfusion, Renal function

## Abstract

**Background:**

Preexisting impaired renal function (IRF) and contrast-induced nephropathy (CIN) after percutaneous coronary intervention (PCI) in patients with ST-segment elevation myocardial infarction (STEMI) are important prognostic parameters, but it is unknown whether delayed PCI is still beneficial for STEMI patients with IRF.

**Methods:**

A retrospective single-center cohort study was performed in 164 patients who presented at least 12 h after symptom onset, and were diagnosed with STEMI and IRF. They were assigned to two groups to receive PCI plus optimal medical therapy (OMT) and OMT alone respectively. Clinical outcomes at 30 days and 1 year were compared between two groups, and hazard ratio for survival was analyzed using Cox regression model. A power analysis demanded 34 patients in each group to produce a power of 90% and a P value of 0.05.

**Results:**

The 30-day mortality was significantly lower in PCI group (n = 126) than in non-PCI group (n = 38) (11.1% versus 28.9%, P = 0.018), while there was no significant difference in the 1-year mortality and incidence of cardiovascular comorbidities between the two groups. Cox regression analysis showed that patients with IRF didn’t benefit from receiving PCI on survival rate (P = 0.267).

**Conclusions:**

Delayed PCI is not beneficial on one-year clinical outcomes for STEMI patients with IRF.

## Introduction

In ST-segment elevation myocardial infarction (STEMI) patients who are transported to the catheterization room within 12 h after the onset of symptoms, percutaneous coronary intervention (PCI) has been proved to increase myocardial salvage, preserve cardiac function, and improve survival [1]. Previous studies have shown that within 2 h of symptom onset, there is a strong linear correlation between mortality and admission time delay in STEMI patients [2, 3], and the mortality increases with longer door-to-balloon time, or first medical contact to PCI time [4, 5]. The 2017 European Society of Cardiology (ESC) guideline recommended a routine primary PCI strategy should be considered in patients presenting late (12–48 h after symptom onset, defined as delayed PCI) after symptom onset (Class IIa, Level of Evidence: B)[6]. A large cohort study from Korea also pointed out that delayed PCI could decrease mortality of the late-presenting STEMI patients [7]. PCI has become the most effective treatment for STEMI patients, but it is unclear whether delayed PCI is still beneficial for STEMI patients with impaired renal function (IRF).

Inflammation is believed to be an important trigger of worse prognosis in STEMI patients with IRF. C-reactive protein to albumin ratio was reported to be higher in patients with renal injury than in patients with normal renal function [8]. In retrospective studies, IRF had a strong adverse impact on the survival of STEMI patients [9, 10], and it is not only an independent predictor of cardiovascular risk, but also contributes to an increased proportion of post-PCI contrast-induced nephropathy (CIN) [11]. Pre-existing IRF could cause contrast metabolism dysfunction after PCI. Evidence shows that CIN associated morbidity and mortality does not decline over time [12], and the incidence of CIN was 5.2% in patients with normal renal function, but the morbidity of CIN was increased up to 26.6% if the patients’ GFR was less than 30 ml/min/1.73m^2^ [13].

Although the 2021 American College of Cardiology Foundation/American Heart Association (ACCF/AHA) guideline for Coronary Artery Revascularization recommended PCI to be performed in patients with STEMI with IRF, the evidence was relatively weak (Level C [14]. Up to now, there are no clear recommendations for PCI treatments in late-coming STEMI patients with IRF. For STEMI patients with IRF and admitted to hospital later than 12 h after symptom onset, it is still controversial whether PCI is more beneficial than optimal medical treatment (OMT) [15, 16], only limited evidence supports delayed PCI [17–19]. Patients with severe IRF were often excluded from large randomized controlled trails (RCTs) because of the risk of adverse ischemic and bleeding events, as well as higher risk of CIN. In real-world practice, a large number of STEMI patients with IRF present longer than 12 h or even 48 h after symptom onset due to inconvenient transportation and unbalanced distribution of medical resources. More evidence needs to be accumulated with regard to whether or not late reperfusion is beneficial in the presence of IRF. Therefore, we performed this study to investigate whether delayed PCI at > 12 h after symptom onset, is superior to OMT alone in the STEMI patients with preexisting IRF.

## Methods

### Study population

**Fig. 1:**
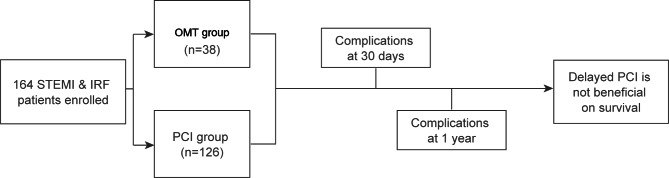
Flowchart of participant inclusion and results. OMT: optimal medical therapy; PCI: percutaneous coronary intervention

A total of 225 STEMI patients with IRF admitted to Zhongshan Hospital from December 2012 to October 2016 were included in this cohort study. STEMI was defined by ischemic symptoms and electrocardiogram changes (i.e. [1] at least two contiguous leads with ST-segment elevation ≥ 2.5 mm in men < 40 years, ≥ 2 mm in men ≥ 40 years, or ≥ 1.5 mm in women in leads V2–V3 and/or ≥ 1 mm in the other leads; [2] ST-segment depression in leads V1–V3 with positive terminal T-wave; [3] concomitant ST-segment elevation ≥ 0.5 mm recorded in leads V7–V9). IRF was defined as decreased estimated glomerular filtration rate (eGFR) level < 60 ml/min/1.73m^2^, calculated using the simplified MDRD formula (eGFR = 186 × creatinine ^-1.154^ × age^-0.203^ × 0.742 (for female only)). We excluded 45 patients who were admitted to hospital within 12 h from symptom onset and 16 patients missing important data about revascularization. Finally, 164 patients were included in the analysis (Fig. 1).

### Treatment

All patients were required to take an 18-lead electrocardiogram (ECG) examination after admission, and echocardiography were recommended to exam after 30 days and 1 year. Two-dimensional echocardiography was performed by using a Philips IE33 ultrasound system to measure the left ventricular end-diastolic diameter (LVEDd) and left ventricular ejection fraction (LVEF) by modified Simpson method.

According to their evaluation of clinical symptoms and ECG results, patients received different revascularization therapies. Revascularization therapy was defined as OMT (including the use of either intravenous fibrinolysis) or intended primary PCI (coronary angiography was performed with an intent to perform PCI). Then the patients were divided into a non-PCI group (n = 38) and a PCI group (n = 126) to assess the effect of these different therapies on clinical outcomes.

Patients younger than 75 years old of the both groups received dual antiplatelet therapy, which was initiated with a loading dose (300 mg of aspirin and 180 mg of ticagrelor), followed by ongoing treatment with aspirin (100 mg/day) and ticagrelor (180 mg/day). Patients over 75 years old received 300 mg of aspirin and 300 mg clopidogrel for loading dose, and ongoing treatment was aspirin (100 mg/day) and clopidogrel (75 mg/day). Other guideline-based medical therapy included use of β-blockers, angiotensin-converting enzyme inhibitors or angiotensin receptor blockers, and statins.

In the PCI group, all PCI procedures were performed by two experienced physicians who had been certified for coronary intervention. The Seldinger technique was employed for PCI. Whether use of GPIIb/IIIa antagonist therapy and an aspiration catheter was depended on the thrombus burden. Subcutaneous injection of low molecular weight heparin was started 3 h after PCI surgery, once every 12 h and 3000 IU each time for 3–5 days. Aspirin (100 mg/day) and ticagrelor (180 mg/day) were initiated from 24 h after PCI.

### Data collection

The following data were collected: Patients’ onset to hospital time, Killip class, systolic blood pressure (SBP), diastolic blood pressure (DBP), and history of smoking, hyperlipidemia, hypertension and diabetes. Levels of cardiac troponin T (cTnT), creatinine, uric acid, glucose, CK-MB and NT-proBNP were acquired through test of serum samples after admission. In hospital medications including intra-aortic balloon pumping (IABP) and extra-corporeal membrane oxygenation (ECMO), cardio pulmonary resuscitation (CPR), pacemaker, vasoactive agents and intravenous diuretics usage were also recorded.

### Definitions and endpoints

The primary end point was death from any cause in 30 days. The secondary end point consisted of all-cause death in 1-year follow up and occurrence of cardiovascular comorbidities, including congestive heart failure, unstable angina pectoris, malignant arrhythmia (which included ventricular fibrillation, ventricular tachycardia and paroxysmal supraventricular tachycardia), cardiac rupture and nonfatal target vessel re-infarction (which was defined as ST-elevation>1 mm recurs or new pathognomonic Q waves appear in at least two contiguous leads and associated with ischemic symptoms) [20]. Clinical outcomes were assessed both at 30 days and up to one year after the onset of STEMI.

### Data analysis

Data are presented as mean ± standard deviation (SD) for normally distributed continuous variables, and were compared by the t-test, while categorical data were expressed as numbers and proportions and were compared by the chi-square test or Fisher’s exact test. Cox proportional hazards regression models were created to evaluate the relation of treatments and 1-year mortality occurrence, adjusting for age, gender, eGFR level, uric acid level, PCI or not, smoking history, hyperlipidemia history, hypertension history and diabetes history. Killip class, SBP and NT-proBNP were considered as mediating variables and were excluded from the regression analysis. All tests were 2-sided and *P* < 0.05 was considered statistically significant. Analyses were performed with SPSS 25.0 (SPSS Inc., Chicago, IL, USA) and R software (http://www.R-project.org/). A power analysis demanded that 34 patients in each group to produce a power of 90% and a P value of 0.05.

## Results

### Baseline characteristics

164 STEMI patients with IRF and presented later than 12 h were divided into non-PCI group and PCI group according to their revascularization methods. Baseline characteristics of the two groups are listed in Table 1. The non-PCI group consisted of 38 patients with the average age of 67.2 years and the PCI group contained 126 patients with the average age of 65.0 years.

**Table 1:** Clinical characteristics at baseline

	Non-PCI (n = 38)	PCI (n = 126)	*P* value
Age, year	67.2 ± 14.8	65.0 ± 13.5	0.237
Male	22 (57.8)	114 (90.4)	< 0.001
Onset to hospital time, hours	57.0 ± 59.8	64.5 ± 76.2	0.574
Killip class			0.003
1	23 (60.5)	83 (65.9)	
2	9 (23.7)	33 (26.2)	
3	4 (10.5)	5 (4.0)	
4	2 (5.3)	5 (4.0)	
cTnT, µg/L	1198. 92 ± 777.63	1120.13 ± 743.78	0.348
SBP, mmHg	129.8 ± 30.8	128.6 ± 25.5	0.830
DBP, mmHg	74.7 ± 16.3	74.5 ± 14.10	0.956
Creatinine, umol/L	147.1 ± 61.7	133.6 ± 51.9	0.180
eGFR, mL/min/1.73m^2^	47.1 ± 15.7	48.6 ± 13.9	0.650
Uric acid, umol/L	449.2 ± 122.9	427.3 ± 125.2	0.350
Fasting glucose, mmol/L	7.0 ± 3.5	7.6 ± 3.7	0.452
C-reactive protein, mg/L	20.74 ± 32.39	18.51 ± 34.92	0.484
CK-MB, IU/L	87.5 ± 120.3	91.8 ± 120.2	0.846
NT-proBNP, pg/mL	8039.9 ± 7482.1	5069.9 ± 6552.3	0.155
Smoker	16 (42.2)	77 (61.1)	0.147
Hyperlipidemia	2 (5.3)	16 (12.7)	0.249
Hypertension	19 (50.0)	68 (54.0)	0.052
Diabetes	5 (13.2)	18 (14.3)	0.223
LVEDd, mm	49.2 ± 5.5	49.30 ± 4.9	0.938
LVEF, %	51.8 ± 10.3	53.30 ± 10.6	0.432

Values are mean ± SD and n (%)

PCI = percutaneous coronary intervention, cTnT = cardiac troponin T, SBP = systolic blood pressure, DBP = diastolic blood pressure, eGFR = estimated glomerular filtration rate, LVEDd = left ventricular end diastolic diameter, LVEF = left ventricular ejection fraction

There were no significant difference between the non-PCI group and the PCI group on all baseline parameters except that the non-PCI group had lower male proportion (57.8% vs. 90.4%).

### Similar 1-year complications and mortality incidence in the two groups

As shown in Table 2, although patients in the non-PCI group had significantly higher mortality than the PCI group in the first 30 days (28.9% vs. 11.1%, *P* = 0.018), there was no significant difference in the 1-year all-cause mortality (44.7% vs. 40.4%, *P* = 0.708). During admission, there was also no difference in the frequency of use of IABP and ECMO, CPR, pacemaker, vasoactive agents, diuretics and morbidity of CIN between the two groups. Patients in the non-PCI group had higher rate of unstable angina pectoris (7.9% vs. 0.0%, P = 0.012) at 30 days after symptom onset, but there was no significant difference between the two groups with regard to incidence of heart failure, malignant arrhythmia, cardiac rupture, nonfatal re-infarction, LVEDd and LVEF. At 1-year follow up, the non-PCI group still exhibited higher rate of unstable angina pectoris (7.9% vs. 0.0%, P = 0.012), while no significant difference in risk of other comorbidities and echocardiographic parameters including LVEDd and LVEF (Table 2).

**Table 2:** Comparisons of latecomer patients according to their revascularization therapies

	Non-PCI (n = 38)	PCI (n = 126)	*P* value
In hospital medications			
IABP + ECMO	1 (2.6)	10 (7.9)	0.463
CPR	0 (0.0)	2 (1.6)	0.863
Pacemaker	0 (0.0)	3 (2.4)	0.331
Vasoactive agents	16 (42.1)	41 (32.5)	0.332
Diuretics	24 (63.2)	72 (57.1)	0.029
CIN	0(0.0)	0(0.0)	NA
Complications at 30 days			
All-cause death	11 (28.9)	14 (11.1)	0.018
Heart failure	3 (7.9)	6 (4.8)	0.457
Unstable angina pectoris	3 (7.9)	0 (0.0)	0.012
Malignant arrhythmia	0 (0.0)	0 (0.0)	NA
Cardiac rupture	0 (0.0)	0 (0.0)	NA
Re-infarction	0 (0.0)	0 (0.0)	NA
LVEDd, mm	49.0 ± 8.3	53.6 ± 8.0	0.248
LVEF, %	53.3 ± 15.3	51.5 ± 11.8	0.219
Complications at 1 year			
All-cause death	17 (44.7)	51 (40.4)	0.708
Heart failure	6 (15.8)	9 (7.1)	0.116
Unstable angina pectoris	3 (7.9)	0 (0.0)	0.012
Malignant arrhythmia	1 (2.6)	0 (0.0)	0.232
Cardiac rupture	0 (0.0)	0(0.0)	NA
Re-infarction	0 (0.0)	0(0.0)	NA
LVEDd, mm	47.3 ± 7.5	51.8 ± 6. 1	0.281
LVEF, %	53.8 ± 12.4	56.4 ± 10.0	0.073

Values are mean ± SD and n (%)

PCI = percutaneous coronary intervention, IABP = intra-aortic balloon pumping, ECMO = extra-corporeal membrane oxygenation, CPR = cardio pulmonary resuscitation, CIN = contrast induced-nephropathy; LVEDd = left ventricular end diastolic diameter, LVEF = left ventricular ejection fraction

### PCI had no improvement on the 1-year clinical outcomes

In the multivariate regression analysis, after adjusting for age, gender, creatinine level, uric acid level, PCI or not, smoking history, hyperlipidemia history, hypertension history and diabetes history, we found that none of the variables had an independent influence on the 1-year survival of patients (Table 3).

**Table 3:** Multivariate analysis of predictors of 1-year survival by Cox proportional hazard model regression

	HR	95%CI	*P* value
**Age, years**	1.011	0.997, 1.026	0.117
**Male**	0.990	0.528, 1.407	0.976
**Creatinine, umol/L**	0.999	0.999, 1.004	0.678
**Uric acid, umol/L**	1.000	0.999, 1.004	0.679
**PCI**	0.746	0.443, 1.268	0.267
**Smoker**	1.095	0.694, 2.513	0.654
**Hyperlipidemia history**	1.218	0.091, 1.603	0.484
**Hypertension history**	0.910	0.591, 1.687	0.604
**Diabetes history**	0.758	0.455, 1.284	0.289

h = hazard ratio, CI = confidence interval, PCI = percutaneous coronary intervention

## Discussion

The recent ESC and AHA guidelines suggested that PCI should be performed in late-presenting patients[14, 16], but there was insufficient evidence about whether PCI could benefit in STEMI latecomers with IRF. In this retrospective analysis, we showed that delayed PCI did not exert significant improvement on 1-year survival in STEMI patients with IRF when compared to OMT alone.

Although “time is myocardium” is a well-known concept in relation to patients with STEMI, a high proportion of eligible patients still do not receive early reperfusion therapy because various reasons such as that the presence of IRF might interfere with decision making [21]. The preexisting IRF is the main independent risk factor for the development of acute kidney injury, and a higher stage of IRF was accompanied by a higher risk [22]. In STEMI patients with normal renal function, several random contrast clinical trials (RCT) showed extremely low mortality after delayed PCI, while OMT alone also had low mortality. The 30-day mortality of OMT patients was only 6.6% in the BRAVE-2 trial [17], and the 1-year mortality in KAMIR trial was 1.1% [23]. Bouisset and colleagues reported that the all-cause mortality of delayed PCI was 2.1% compared to 7.2% of the non-PCI group, indicating a significant benefit from PCI in patients with normal renal function. However, the mortality largely increased when STEMI patients had preexisting IRF. A large cohort study from Malaysia demonstrated STEMI patients with IRF had 4.55 times higher rate of developing in-hospital death compared to patients with normal renal function [24]. Another national study from Australia found worsening IRF severity was independently associated with greater adjusted risk of long-term death (Hazard ratio 4.21, 95% CI 3.7–4.8)[25], while an Italy retrospective cohort study revealed that patients with severe and moderate chronic kidney disease were more likely to develop in-hospital death than non-CKD patients (50% and 19.08% versus 2.93%, P < 0.0001), and the long-term mortality rate increased up to 57.14% and 46.34% versus 8.77%[26]. These findings indicate that IRF would greatly increase mortality even in STEMI patients with onset to hospital time less than 12 h.

It is unclear whether delayed PCI plus OMT is better than OMT alone in STEMI patients with IRF. We found that delayed PCI significantly reduced the 30-day mortality of STEMI patients with IRF, but that benefit could not persist evidenced by no significant difference on 1-year mortality between the two groups. ISCHEMIA-CKD trial enrolled 777 patients with severe or end-stage IRF and moderate or severe ischemia to receive coronary revascularization or initial medical therapy alone. After 2 to 4 years’ follow-up, the results indicated that there were no significant differences between the two groups in the individual end points of death, cardiovascular death, myocardial infarction, unstable angina, or heart failure [27]. The investigators concluded that PCI strategy does not decrease death or ischemic events, does not improve quality of life, but might increase the early need for dialysis. Taken together, it should be cautious to make decision of performing delayed PCI for STEMI patients with IRF. If the IRF could be reversed by OMT, the patients with old myocardial infarction may benefit from time-selective PCI. It is elusive why IRF antagonizes the benefit from PCI. A possible explanation is that deteriorating renal metabolism dysfunction dilutes the benefits of PCI, because besides lipid composition change, the metabolism profiles of fatty acids are altered in IRF patients [28]. In addition, differential expressions of large number of transcripts have been identified through next generation sequencing, and alterations in various signal pathways related to metabolic, apoptotic and other essential biological processes also contribute to the harmful influence of IRF on the benefit from PCI [29, 30]. Moreover, mitochondrial overload also occurs in the impaired kidney, which could result in the dysfunction of renal energy metabolism and tubulointerstitial fibrosis [31] and finally induced dysfunction of multiple organs.

In real-world studies, risk factors should be adjusted before comparison of mortality. It was reported that ischemic symptoms in female patients were more atypical [32], this partially explained the difference of gender proportion in our analysis (57.8% male in the non-PCI group versus 90.4% in the PCI group). Lawesson et al. reported that female gender was independently associated with risk of IRF in patients with STEMI, which seemed to be an important reason why STEMI women have higher mortality than men [33]. Interestingly, we used Cox proportional hazards model and found that male sex was not related to better survival, indicating that although misleading symptoms could lead to delays in presentation time, it would not affect survival in IRF patients.

### Limitations

The main limitation of this study is its observational design. It does not confirm cause and effect, but only describes a statistically significant and independent association between observed clinical outcomes and patients’ management. As a single-center study, the sample size was limited, which may induce inevitable bias. A RCT of larger sample size with more potential confounding factors should be performed further to add more solid evidence to the guidelines.

## Conclusions

Delayed PCI is not beneficial on the clinical outcomes for STEMI patients with IRF.

## Data Availability

The datasets analyzed during the current study are not publicly available due to subsequent analysis on other topics but are available from the corresponding author on reasonable request.

## List of abbreviations

PCI: percutaneous coronary intervention
STEMI: ST-segment elevation myocardial infarction
IRF: impaired renal function
HR: hazard ratios
CIN: contrast-induced nephropathy
eGFR: glomerular filtration rate
LVEDd: left ventricular end-diastolic diameter
LVEF: left ventricular ejection fraction
RCT: randomized controlled trails
IABP: intra-aortic balloon pumping
ECMO: extra-corporeal membrane oxygenation
CPR: cardio pulmonary resuscitation
CKD: chronic kidney disease

## References

[1] Keeley EC, Boura JA, Grines CL (2003). Primary angioplasty versus intravenous thrombolytic therapy for acute myocardial infarction: a quantitative review of 23 randomised trials. Lancet.

[2] Boersma E, Maas AC, Deckers JW, Simoons ML (1996). Early thrombolytic treatment in acute myocardial infarction: reappraisal of the golden hour. Lancet.

[3] Gersh BJ, Stone GW, White HD, Holmes DR (2005). Pharmacological facilitation of primary percutaneous coronary intervention for acute myocardial infarction: is the slope of the curve the shape of the future?. JAMA.

[4] Chandrasekhar J, Marley P, Allada C, McGill D, O’Connor S, Rahman M (2017). Symptom-to-balloon time is a strong predictor of adverse events following primary percutaneous coronary intervention: results from the australian Capital Territory PCI Registry. Heart Lung Circ.

[5] Koul S, Andell P, Martinsson A, Gustav Smith J, van der Pals J, Schersten F (2014). Delay from first medical contact to primary PCI and all-cause mortality: a nationwide study of patients with ST-elevation myocardial infarction. J Am Heart Association.

[6] Neumann FJ, Sousa-Uva M, Ahlsson A, Alfonso F, Banning AP, Benedetto U (2019). 2018 ESC/EACTS guidelines on myocardial revascularization. Eur Heart J.

[7] Bouisset F, Gerbaud E, Bataille V, Coste P, Puymirat E, Belle L (2021). Percutaneous myocardial revascularization in late-presenting patients with STEMI. J Am Coll Cardiol.

[8] Karabağ Y, Çağdaş M, Rencuzogullari I, Karakoyun S, Artaç İ, İliş D (2019). The C-Reactive protein to albumin ratio predicts acute kidney Injury in Patients with ST-Segment Elevation myocardial infarction undergoing primary percutaneous coronary intervention. Heart Lung Circ.

[9] Chiang CY, Huang SC, Chen M, Shih JY, Hong CS, Wu NC (2021). Effects of renal impairment on cardiac remodeling and clinical outcomes after myocardial infarction. Int J Med Sci.

[10] Patel AD, Ibrahim M, Swaminathan RV, Minhas IU, Kim LK, Venkatesh P (2017). Five-year mortality outcomes in patients with chronic kidney disease undergoing percutaneous coronary intervention. Catheter Cardiovasc Interv.

[11] Çınar T, Karabağ Y, Ozan Tanık V, Çağdaş M, Rencüzoğulları İ, Öz A (2020). The investigation of TIMI risk index for prediction of contrast-induced acute kidney injury in patients with ST elevation myocardial infarction. Acta Cardiol.

[12] Lun Z, Liu L, Chen G, Ying M, Liu J, Wang B (2021). The global incidence and mortality of contrast-associated acute kidney injury following coronary angiography: a meta-analysis of 1.2 million patients. J Nephrol.

[13] Tsai TT, Patel UD, Chang TI, Kennedy KF, Masoudi FA, Matheny ME (2014). Contemporary incidence, predictors, and outcomes of acute kidney injury in patients undergoing percutaneous coronary interventions: insights from the NCDR Cath-PCI registry. JACC Cardiovasc interventions.

[14] Lawton JS, Tamis-Holland JE, Bangalore S, Bates ER, Beckie TM, Bischoff JM et al. 2021 ACC/AHA/SCAI Guideline for Coronary Artery Revascularization: A Report of the American College of Cardiology/American Heart Association Joint Committee on Clinical Practice Guidelines. Circulation. 2021:Cir0000000000001038. 10.1161/CIR.0000000000001038 34882435

[15] O’Gara PT, Kushner FG, Ascheim DD, Casey DE, Chung MK, de Lemos JA (2013). 2013 ACCF/AHA guideline for the management of ST-elevation myocardial infarction: executive summary: a report of the American College of Cardiology Foundation/American Heart Association Task Force on Practice Guidelines. Circulation.

[16] Ibanez B, James S, Agewall S, Antunes MJ, Bucciarelli-Ducci C, Bueno H (2018). 2017 ESC Guidelines for the management of acute myocardial infarction in patients presenting with ST-segment elevation: the Task Force for the management of acute myocardial infarction in patients presenting with ST-segment elevation of the European Society of Cardiology (ESC). Eur Heart J.

[17] Schomig A, Mehilli J, Antoniucci D, Ndrepepa G, Markwardt C, Di Pede F (2005). Mechanical reperfusion in patients with acute myocardial infarction presenting more than 12 hours from symptom onset: a randomized controlled trial. JAMA.

[18] Ndrepepa G, Kastrati A, Mehilli J, Antoniucci D, Schomig A (2009). Mechanical reperfusion and long-term mortality in patients with acute myocardial infarction presenting 12 to 48 hours from onset of symptoms. JAMA.

[19] Busk M, Kaltoft A, Nielsen SS, Bottcher M, Rehling M, Thuesen L (2009). Infarct size and myocardial salvage after primary angioplasty in patients presenting with symptoms for < 12 h vs. 12–72 h. Eur Heart J.

[20] Thygesen K, Alpert JS, Jaffe AS, Chaitman BR, Bax JJ, Morrow DA (2018). Fourth Universal Definition of myocardial infarction (2018). Glob Heart.

[21] Shahin M, Obeid S, Hamed L, Templin C, Gamperli O, Nietlispach F (2017). Occurrence and impact of Time Delay to primary percutaneous coronary intervention in patients with ST-Segment Elevation myocardial infarction. Cardiol Res.

[22] Bangalore S, Maron DJ, Fleg JL, O’Brien SM, Herzog CA, Stone GW (2018). International Study of Comparative Health Effectiveness with Medical and Invasive Approaches-Chronic kidney disease (ISCHEMIA-CKD): Rationale and design. Am Heart J.

[23] Sim DS, Jeong MH, Ahn Y, Kim YJ, Chae SC, Hong TJ (2012). Benefit of percutaneous coronary intervention in early latecomers with acute ST-segment elevation myocardial infarction. Am J Cardiol.

[24] Ismail MD, Jalalonmuhali M, Azhari Z, Mariapun J, Lee ZV, Zainal Abidin I (2018). Outcomes of STEMI patients with chronic kidney disease treated with percutaneous coronary intervention: the Malaysian National Cardiovascular Disease database - percutaneous coronary intervention (NCVD-PCI) registry data from 2007 to 2014. BMC Cardiovasc Disord.

[25] Bloom JE, Dinh DT, Noaman S, Martin C, Lim M, Batchelor R (2021). Adverse impact of chronic kidney disease on clinical outcomes following percutaneous coronary intervention. Catheter Cardiovasc Interv.

[26] Peyracchia M, Scacciatella P, Conrotto F, Meynet I, Biava LM, Budano C (2018). Impact of chronic kidney disease on mortality in patients with ST-segment elevation myocardial infarction treated with primary percutaneous coronary intervention. A long-term single-center mortality study. Minerva Cardioangiol.

[27] Levine GN, Umair Khalid M (2020). ISCHEMIA-CKD: contemporary randomized Clinical Data at Last. Circulation.

[28] Afshinnia F, Rajendiran TM, Soni T, Byun J, Wernisch S, Sas KM (2018). Impaired β-Oxidation and altered complex lipid fatty acid partitioning with advancing CKD. J Am Soc Nephrol.

[29] Liu Y, Liu B, Liu Y, Chen S, Yang J, Liu J (2019). MicroRNA expression profile by next-generation sequencing in a novel rat model of contrast-induced acute kidney injury. Annals of translational medicine.

[30] Chen G, Liu B, Chen S, Li H, Liu J, Mai Z (2021). Novel biomarkers for post-contrast acute kidney injury identified from long non-coding RNA expression profiles. Int J Biol Sci.

[31] Kang HM, Ahn SH, Choi P, Ko YA, Han SH, Chinga F (2015). Defective fatty acid oxidation in renal tubular epithelial cells has a key role in kidney fibrosis development. Nat Med.

[32] Lichtman JH, Leifheit EC, Safdar B, Bao H, Krumholz HM, Lorenze NP (2018). Sex differences in the presentation and perception of symptoms among young patients with myocardial infarction: evidence from the VIRGO Study (Variation in Recovery: role of gender on outcomes of young AMI patients). Circulation.

[33] Sederholm Lawesson S, Alfredsson J, Szummer K, Fredrikson M, Swahn E (2015). Prevalence and prognostic impact of chronic kidney disease in STEMI from a gender perspective: data from the SWEDEHEART register, a large swedish prospective cohort. BMJ open.

